# Non-Invasive Assessment of Breast Cancer Molecular Subtypes with Multiparametric Magnetic Resonance Imaging Radiomics

**DOI:** 10.3390/jcm9061853

**Published:** 2020-06-14

**Authors:** Doris Leithner, Marius E. Mayerhoefer, Danny F. Martinez, Maxine S. Jochelson, Elizabeth A. Morris, Sunitha B. Thakur, Katja Pinker

**Affiliations:** 1 Department of Radiology, Memorial Sloan Kettering Cancer Center, New York, NY 10065, USA; doris.leithner@gmail.com (D.L.); martind4@mskcc.org (D.F.M.); jochelsm@mskcc.org (M.S.J.); morrise@mskcc.rog (E.A.M.); thakurs@mskcc.org (S.B.T.); pinkerdk@mskcc.org (K.P.); 2 Department of Biomedical Imaging and Image-guided Therapy, Medical University of Vienna, 1090 Vienna, Austria; 3 Department of Medical Physics, Memorial Sloan Kettering Cancer Center, New York, NY 10065, USA

**Keywords:** radiomics, diffusion-weighted, magnetic resonance imaging, breast cancer, molecular subtypes

## Abstract

We evaluated the performance of radiomics and artificial intelligence (AI) from multiparametric magnetic resonance imaging (MRI) for the assessment of breast cancer molecular subtypes. Ninety-one breast cancer patients who underwent 3T dynamic contrast-enhanced (DCE) MRI and diffusion-weighted imaging (DWI) with apparent diffusion coefficient (ADC) mapping were included retrospectively. Radiomic features were extracted from manually drawn regions of interest (n = 704 features per lesion) on initial DCE-MRI and ADC maps. The ten best features for subtype separation were selected using probability of error and average correlation coefficients. For pairwise comparisons with >20 patients in each group, a multi-layer perceptron feed-forward artificial neural network (MLP-ANN) was used (70% of cases for training, 30%, for validation, five times each). For all other separations, linear discriminant analysis (LDA) and leave-one-out cross-validation were applied. Histopathology served as the reference standard. MLP-ANN yielded an overall median area under the receiver-operating-characteristic curve (AUC) of 0.86 (0.77–0.92) for the separation of triple negative (TN) from other cancers. The separation of luminal A and TN cancers yielded an overall median AUC of 0.8 (0.75–0.83). Radiomics and AI from multiparametric MRI may aid in the non-invasive differentiation of TN and luminal A breast cancers from other subtypes.

## 1. Introduction

Breast cancer therapies are driven by tumour biology with four main intrinsic molecular subtypes of breast cancer that show substantial differences in phenotype, prognosis, treatment response, and survival [1,2,3,4]: luminal A, luminal B, human epidermal growth factor receptor 2 (HER2)-enriched, and triple negative (TN) [5,6]. In clinical practice, the assessment of molecular subtypes for treatment planning is mostly performed from immunohistochemical surrogates from invasive tissue sampling [3]. Nevertheless, this approach is potentially flawed, as a biopsy can only deliver a small amount of tissue, while genetic heterogeneity within a single tumour is a major cause of therapy resistance and treatment failure [7,8,9,10]. Furthermore, genetic profiling is performed at a single time point, although tumour biology might potentially change over time and with treatment [11]. The rapidly evolving field of radiomics is based on the assumption that tumour biology is reflected in microstructural patterns within medical imaging, some of which the human eye cannot perceive. Recent advances in biomedical image acquisition, high-throughput methods for analysis, and the application of artificial intelligence (AI) have improved the quantification of these radiomic features, which may non-invasively provide information for a given tumour in its entirety. In this context, radiomics coupled with AI has the potential to improve patient stratification, treatment planning, and therapy monitoring, and may be combined with clinical and genomic data to achieve the overarching goal of precision medicine. Previous radiomics studies in breast imaging have primarily focused on features derived from dynamic contrast-enhanced MRI (DCE-MRI), with promising results for the non-invasive identification of different types of breast cancer [12,13,14]. Although multiparametric MRI, including DCE-MRI and diffusion-weighted imaging (DWI), is widely recommended, [15] data are scarce on DWI radiomic signatures and their utility in this context [16,17]. Few prior studies have evaluated combined multiparametric MRI with DCE-MRI and DWI radiomics signatures in the breast; however, the generalisation of these findings is limited due to the use of different imaging protocols and scanners [18], the investigation of histogram features only [19], and the lack of advanced artificial intelligence-based machine learning algorithms for analysis [18,19].

We hypothesised that microstructural differences between breast cancer subtypes exist that can be captured with multiparametric MRI radiomics and classified using AI. Therefore, the purpose of this study was to evaluate the performance of radiomics and AI from multiparametric MRI for the assessment of breast cancer receptor status and molecular subtypes.

## 2. Material and Methods

This retrospective single-institution study is in accordance with Health Insurance Portability and Accountability Act guidelines and was approved by the Institutional Review Board with a waiver for written informed consent.

### 2.1. Patients

A database search was conducted for patients who underwent multiparametric MRI of the breast including DCE-MRI and DWI between January 2011 and January 2013, and who met the following inclusion criteria: histopathologically verified breast cancer; patient age, 18 years or older; and lesion size, greater than 1 cm on DCE-MRI to reduce the influence of the partial volume effect on the radiomics analysis. The exclusion criteria were as follows: other types of breast cancer besides invasive ductal carcinoma, a personal history of other cancers, a high risk status, prior systemic cancer treatment, pregnancy or breast-feeding at the time of MRI, poor MR image quality, and artefacts leading to signal distortions in the tumour area, as assessed by a board-certified radiologist specialising in breast imaging. Thus, 91 patients were included in this study.

### 2.2. MR Imaging

All MRI examinations were performed using a 3 Tesla scanner (Discovery MR750; GE Healthcare, Milwaukee, WI, USA) with a body coil as the transmitter and a dedicated 16-channel phased-array breast coil (Sentinelle Vanguard, Toronto, ON, Canada) as the receiver. The state-of-the-art MRI protocol consisted of the following pulse sequences: a T2-weighted imaging sequence with and without fat suppression (fast spin-echo sequence; TR/TE, 4.384/102 ms; auto flip angle, 111°; matrix size, 288 × 224; FOV, 30 cm; slice thickness, 3 mm; NEX, 2; acquisition time, approximately 3.5 min); a 2D, single-shot, dual spin echo-planar DWI sequence (TR, 6.000 ms; minimum TE; flip angle, 90°; acquisition matrix, 98 × 98 or 128 × 128; reconstructed matrix, 256 × 256; FOV, 28–38 cm; slice thickness, 4 or 5 mm; NEX, 3; slice gap, 0–1 mm; fat suppression, enhanced; parallel imaging, ASSET; acquisition time, approximately 2 min for two b-values of 0 and 1000); and a DCE-MRI sequence with and without fat suppression (3D T1-weighted gradient echo VIBRANT sequence; TR/TE, 4.3/2.1 ms; flip angle, 10°; matrix size, 320 × 192; FOV, 30 cm; 1.0 mm slice thickness; NEX, 1; acquisition time, approximately 1 min). The DCE-MRI series were acquired before and at three points at 60 s intervals after a standard dose (0.1 mmol/kg body weight) of gadopentetate dimeglumine (Magnevist; Bayer HealthCare, Hanover, NJ, USA) was injected intravenously as a bolus, followed by a saline flush.

### 2.3. Radiomics Analysis

The first post-contrast T1-weighted sequence and the apparent diffusion coefficient (ADC) map were used for radiomics analysis. Feature extraction was performed semi-automatically using the open-source software MaZda 4.6 (http://www.eletel.p.lodz.pl/programy/mazda/). Two radiologists with 13 and four years of experience analysed images in consensus. A single two-dimensional region of interest (ROI) covering the whole tumour on the DCE-MRI section depicting the largest transaxial lesion diameter was drawn manually. Texture parameters were extracted from the DCE images on the one hand, and from the ADC maps on the other hand. Due to the better contrast between the lesion and the surrounding tissue, the ROI was drawn on the DWI images but then copied to the ADC map; the texture features were then extracted from this ROI on the ADC map (i.e., not from the DWI images). A minimum distance of 2 mm was kept from any biopsy markers present. Artefacts were excluded from segmentation. Grey-level normalisation was performed (dynamics, µ ± 3σ; µ, grey-level mean; σ, grey-level standard deviation) to reduce contrast and brightness variations that might impair texture feature quantification [20]. All the radiomic features that MaZda was capable of calculating were included, resulting in a total number of 704 features per lesion. Features derived from various classes, such as the first-order histogram (*n* = 18), lesion geometry (*n* = 146), absolute gradient (*n* = 10), autoregressive model (*n* = 10), co-occurrence matrix (n = 440), run-length matrix (n = 40), and discrete Haar wavelet transform (*n* = 40) were calculated (see http://www.eletel.p.lodz.pl/programy/mazda/download/FeaturerList.pdf for full feature list). Radiomic features represent a multitude of tissue characteristics such as shape, heterogeneity, intensity, and local interactions between pixels. The total time of lesion segmentation and radiomics analysis was approximately 5 min per patient.

### 2.4. Statistical Analysis

Out of the large number of features obtained, the five most relevant features for the differentiation of molecular subtypes were selected separately for each technique (i.e., DCE-MRI and DWI). For this study, the minimisation of the probability of error and average correlation coefficients (POE + ACC) were used for feature selection. Contrary to other criteria such as Fisher coefficients, POE + ACC takes interrelationships between features into account with the aim of reducing data redundancy [21]. Feature selection was performed once across the training dataset prior to radiomics-based classification. Histopathology served as the standard of reference.

To differentiate between two groups with more than twenty patients each, a multi-layer perceptron feed-forward artificial neural network (MLP-ANN), which is based on a back-propagation learning algorithm, was used. For each pairwise classification, 70% of the respective cohort were used for training and 30%, for validation. Classification was performed five times for each pairwise comparison, as the starting point of an MLP-ANN is an initial guess at the weights of single features. For each repetition of the classification step, patients were randomly assigned anew to the training or validation dataset. A minimum of one hidden layer with a minimum of three neurons per hidden layer was used for the neural network. Areas under the receiver operating characteristic (ROC) curves (AUCs), as well as the diagnostic accuracies for the training and validation datasets, were calculated. The MLP-ANN was applied using SPSS 24.0 (IBM Corp., Armonk, NY, USA).

For the separation of two groups with fewer than twenty patients each, linear discriminant analysis (LDA) was used for feature reduction, producing so-called “most discriminating features” (MDF). Hereafter, leave-one-out cross validation (LOOCV), as implemented in the B11 module of the MaZda 4.6 software, was applied for radiomics-based pairwise classification, i.e., training was performed using all patients except one, excluding information from the held-out patient, and testing was conducted on the remaining patient. This process was repeated n times, with n being the number of subjects in each comparison.

### 2.5. Histopathological Analysis

Tumour histology, tumour and nuclear grade, and immunohistochemical status including oestrogen receptor, progesterone receptor, and HER2 status were derived from final histopathological results from surgical tumour specimens. Oestrogen or progesterone receptor-positive tumours with over 1% staining were classified as hormone receptor (HR)-positive. Tumours were classified as luminal A for HR-positive and HER2-negative, luminal B for HR-positive and HER2-positive, HER2-enriched for HR-negative and HER2-positive, and TN for HR- and HER2-negative [22]. In the case of equivocal HER2 status, lesions were additionally evaluated using fluorescence in situ hybridisation and classified as positive when gene amplification was detected.

## 3. Results

Of the 91 treatment-naïve, biopsy-proven breast cancers, 57 were HR positive (62.6%). Forty-nine cancers were classified as luminal A (53.8%), eight as luminal B (8.8%), 11 as HER2-enriched (12.1%), and 23 as TN (25.3%). There were 70 mass lesions and 21 non-mass enhancing lesions on DCE-MRI. The mean lesion size was 3.5 ± 2.3 cm (range, 1–16.6 cm). The mean patient age was 48 ± 9.7 years (range, 27–68 years).

In four pairwise classifications, the group sizes were large enough, i.e., there were over 20 patients in each group, and thus, the MLP-ANN was used, while for all other analyses, LDA and LOOCV were applied. AUCs higher than 0.8 and accuracies above 80% were considered to be sufficient for possible clinical application in terms of the assessment of molecular subtypes and HR status.

**Figure 1:**
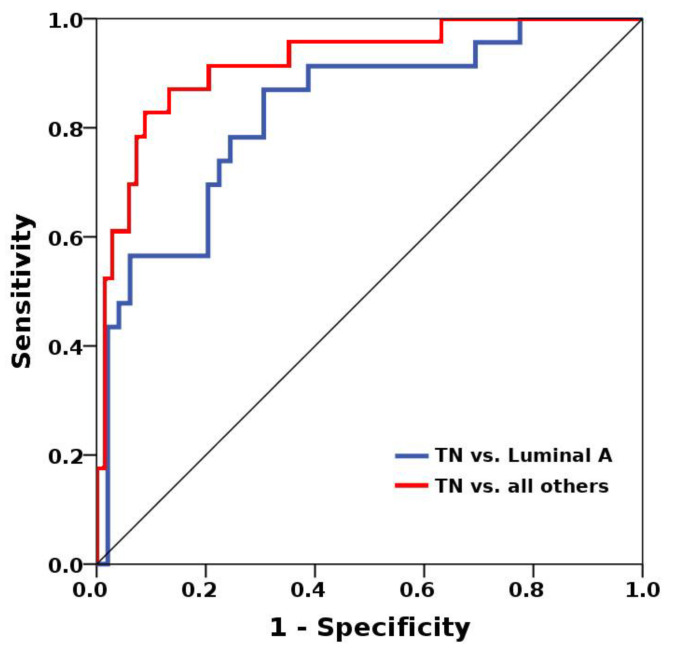
Multi-layer perceptron feed-forward artificial neural network (MLP-ANN)-based separation of luminal A and triple negative (TN) cancers yielded an overall median area under the receiver operating characteristic (ROC) curve (AUC) of 0.8 (0.75–0.83), with median accuracies of 74% in the training dataset and 68.2% in the validation dataset (blue ROC curve). The separation of TN from all other cancers was even more successful, with an overall median AUC of 0.86 (0.77–0.92), with median accuracies of 85.9% in the training dataset and 85.2% in the validation dataset (red ROC curve).

**Figure 2:**
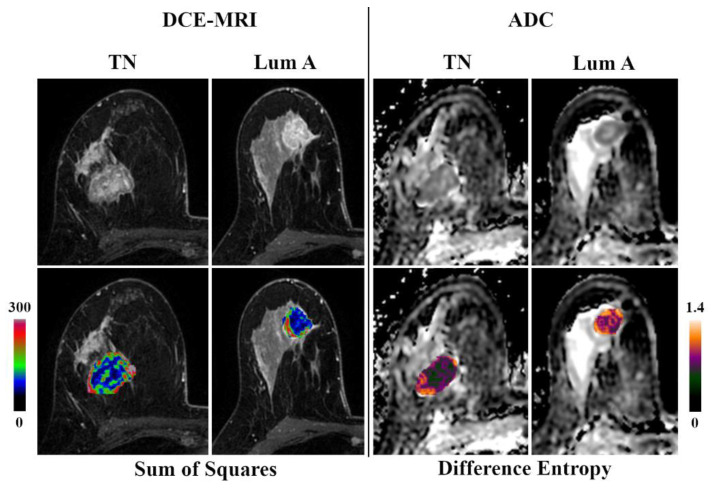
Original DCE-MRI images/ADC maps and corresponding color-coded feature maps as overlays of the tumor area of triple negative (TN) and luminal A breast cancer.

Best results in terms of AUCs were achieved for the differentiation of TN cancers from other groups and luminal A cancers from other groups (Table 1). The MLP-ANN yielded an overall median AUC of 0.86 (0.77–0.92) for the separation of TN from all other cancers (Figure 1), with median accuracies of 85.9% in the training dataset and 85.2% in the validation dataset. For the separation of these subtypes, a balanced mix of features from different categories such as histogram, texture, shape-based, and model-based, proved to be of importance (Table 2). The discrimination of luminal A from TN cancers yielded an overall median AUC of 0.8 (0.75–0.83), with median accuracies of 74% in the training dataset and 68.2% in the validation dataset. Again, a balanced mix of features from different feature groups was useful for lesion classification. Hence, luminal A and TN cancers seem to carry distinct radiomic characteristics that enable their separation from other breast cancers (Figure 2). All other AUCs were below 0.8, and diagnostic accuracies, below 80% (Table 3).

## 4. Discussion

In this study, we assessed the performance of multiparametric MRI-based radiomics in conjunction with AI for the assessment of breast cancer receptor status and molecular subtypes. Our results indicate that radiomics signatures derived from multiparametric MRI enable the determination of certain treatment-naïve molecular breast cancer subtypes with high accuracy. Although radiomics and AI are unlikely to replace invasive tissue sampling, multiparametric radiomics imaging biomarkers could potentially serve as auxiliary parameters and a non-invasive method to derive prognostic and predictive information from the entire tumour before and during treatment.

In the present study, the best results in terms of accuracies were achieved for the radiomics-based separation of TN from all other cancers, as well as luminal A and TN cancers (AUCs, 0.80 and 0.76). TN is the most aggressive type of cancer and carries a worse prognosis in comparison to the other subtypes. While patients with luminal A cancers may be offered endocrine therapy in addition to surgery and radiation treatment, and patients with HER2-positive cancers may receive additional targeted treatment with monoclonal antibodies, patients with TN cancers currently have no available targeted treatment [4]. After additional validation, these specific results of our study might have direct clinical consequences, as they might prevent the exclusion of patients from adequate therapy when a heterogeneous tumour is present. The relevant information of tumour heterogeneity and the expression of HER2 or HR might otherwise go unnoticed, as biopsy can only deliver a small amount of tissue, and after neoadjuvant chemotherapy, no cancerous tissue for analysis might be available anymore.

Previous radiomics studies in breast imaging have primarily focused on signatures extracted from DCE-MRI and their utility e.g., for the separation of benign and malignant lesions [23,24], the prediction of treatment response [25], and the separation of molecular subtypes [26,27], with mixed results, which might be attributed to heterogeneity in scanners, sequences, and features. Holli-Helenius et al. reported AUC values of 0.83–0.88 for the differentiation of luminal A from luminal B cancers in a small patient collective using only co-occurrence matrix features [28], while in the present study, the separation of luminal A from TN tumours based on features from different groups was more successful. Surprisingly, in times of the controversy regarding the application of gadolinium-based contrast agents [29,30], data are scarce on radiomic features derived from DWI, with results from a single study suggesting that they might be useful for the assessment of breast cancer subtypes [31].

Multiparametric breast MRI combining both DCE and DWI is increasingly used in clinical routine practice and is recommended to improve diagnostic accuracy, tumour characterisation, and response assessment [15]. Although we acknowledge that, to date, it is not possible to adequately identify molecular subtypes using imaging alone, initial hints at underlying tumour biology may be found, such as rapid wash-out, rim enhancement, and a higher ADC in TN tumours [32]. These imaging patterns can also be easily captured and quantified with radiomics analysis [33]. In the pairwise comparisons achieving an AUC >80% in our study, a very balanced mix of histogram, texture, shape-based, and model-based features seemed to be of importance for radiomics-based lesion classification, which emphasises the complementary value of the combination of MRI sequences to capture different functional aspects of tumour biology (e.g., diffusivity and perfusion).

In the first study to investigate the value of multiparametric MRI radiomic signatures in this context, Sun et al. achieved excellent accuracies of up to 97.7% for the separation of subtypes in 107 patients [18]. Notably, the authors relied on a small number of features and used Fisher discriminant analysis in a less advanced machine learning approach than the MLP-ANN used in the present study. Meanwhile, Xie et al. evaluated histogram features derived from multiparametric MRI for the differentiation of TN cancers from other subtypes in 134 patients, yielding AUCs of up to 0.76 [19]. These results are in good agreement with our findings; nevertheless, their applicability is limited, as histogram features alone cannot provide true textural information in terms of the relationships between single pixels/voxels of a tumour [21]. Clearly, rigorous standardisation in terms of image acquisition and analysis across institutions, or the use of deep learning neural networks in very large heterogeneous datasets, will be essential to enable the widespread application of radiomics in clinical practice. To our knowledge, this is the first study to examine the utility of combined DCE-MRI and DWI radiomic signatures for the differentiation between breast cancers of different molecular subtypes/receptor status using a strictly homogeneous imaging protocol and an advanced neural network.

This study has limitations beyond its retrospective single-centre approach, which have to be acknowledged. First, all tumours were segmented manually and on the slice with the largest diameter only, which might not be feasible in the analysis of very large datasets. Furthermore, this approach might not capture tumour heterogeneity in its entirety, and it is therefore quite possible that better results might have been achieved with 3D texture features. Second, all ROIs were defined on high b-value DWI and transferred to the ADC map, although tumour segmentation directly on ADC maps has previously shown to be slightly beneficial for radiomics analysis [31]. However, some lesions could not be confidently identified based on ADC maps alone and thus this approach was chosen. Third, although MLP networks are well-established powerful machine learning algorihms, even more advanced deep learning techniques such as convolutional neural networks with larger numbers of hidden layers and interconnections between neurons may have performed better. However, the complexity of these advanced algorithms used on a relatively small patient collective such as our own would have increased the possibility of overfitting and reduced generalisability. MLP networks are even increasingly recommended as they are high-performing, reliable, and robust models for classification tasks [34]. Finally, the same patient cohort as in a previous study was used [31]; however, there were major differences from a methodological point of view: (1) All classifications in the present study were based on a mix of DCE-MRI and DWI features, i.e., unlike in the previous study, DWI features were never used on their own, and only a subset of DWI features was used. This combined DCE + DWI approach, which aims for a more holistic representation of tumour biology by taking both tissue perfusion and diffusivity into account, therefore represents an extension of our previously published results. (2) The main results of the present study are based on a machine learning algorithm—an MLP neural network—that is clearly more advanced than the approach used in our previous study. The MLP-ANN requires the true separation of training and validation data and therefore provides a more realistic assessment of model performance.

In conclusion, the results of study indicate the potential of radiomic signatures from multiparametric MRI with DCE-MRI and DWI to allow the separation of aggressive and non-aggressive subtypes (i.e., TN and luminal A) with high accuracy. Radiomic characteristics derived from multiparametric MRI might have the potential to yield imaging biomarkers and thus may be used to monitor tumour biology changes during treatment and, hence, provide decision support. Further studies with larger patient numbers using even more advanced neural networks are warranted to validate these initial findings and fully elucidate the potential of radiomics and AI in this context.

## Figures and Tables

**Table 1:** Mean classification accuracies for radiomics data.

	Training Accuracy Median (Range) %	Test Accuracy Median (Range) %	AUC Median (Range) %
Luminal A vs. TN	74 (70–86)	68.2 (63.6–81.8)	0.8 (0.75–0.83)
Luminal A vs. all others	65.6 (62.5–78.6)	66.7 (59.3–74.1)	0.72 (0.7–0.74)
TN vs. all others	85.9 (78.1–91.3)	85.2 (85.2–90.9)	0.86 (0.77–0.92)
HR+ vs. HR−	64.7 (63.2–80.9)	60 (52.2–82.6)	0.69 (0.63–0.89)

Note: AUC, area under the curve; HR, hormone receptor; TN, triple negative.

**Table 2:** Selected feature sets for pairwise classifications with areas under the curve higher than 0.8.

	TN vs. All Others	Luminal A vs. TN
**DCE-MRI**	Sum of squares	Sum of squares
	Vertical coordinate of gravity centre	Theta 2
	Vertical second order moment of inertia	GeoFmax/GeoFmin
	Theta 2	Danielsson ratio
	Histogram’s variance	Histogram’s variance
**ADC map**	Difference entropy	Sum of squares
	Sum average	Difference variance
	Absolute gradient skewness	Theta 2
	Difference variance	Difference variance
	Sum of squares	Histogram’s skewness

Note: ADC, apparent diffusion coefficient; DCE-MRI, dynamic contrast-enhanced MRI; TN, triple negative.

**Table 3:** Results of group-wise radiomic feature-based classifications for molecular breast cancer subtypes using linear discriminant analysis and leave-one-out cross validation.

	HER2 Negative	Luminal A	Luminal B	HER2-Enriched	TN	All Others
HER2 positive	67.7%	-	-	-	-	67.7%
Luminal A	-	-	52.6%	56.7%	-	-
Luminal B	-	52.6%	-	57.9%	38.7%	58.2%
HER2-enriched	-	56.7%	57.9%	-	70.3%	54.9%
TN	-	-	38.7%	70.3%	-	-
All others	67.7%	-	58.2%	54.9%	-	-

Note: HER2, human epidermal growth factor receptor 2; TN, triple negative.
